# Supplementary material for: Splice-Junction-Based Mapping of Alternative Isoforms in the Human Proteome
Source: Cell Rep. Author manuscript; Available in PMC 2020 Jan 15. (PMC6961840; doi:10.1016/j.celrep.2019.11.026)
Supplement: 2 [file NIHMS1546469-supplement-2.zip › DF1/PXD006675/RightVentricle/RightVentricle_48_THBS1_NALWHTGNTPGQVMMSLMLWTSVAPSSSTPK.pdf]

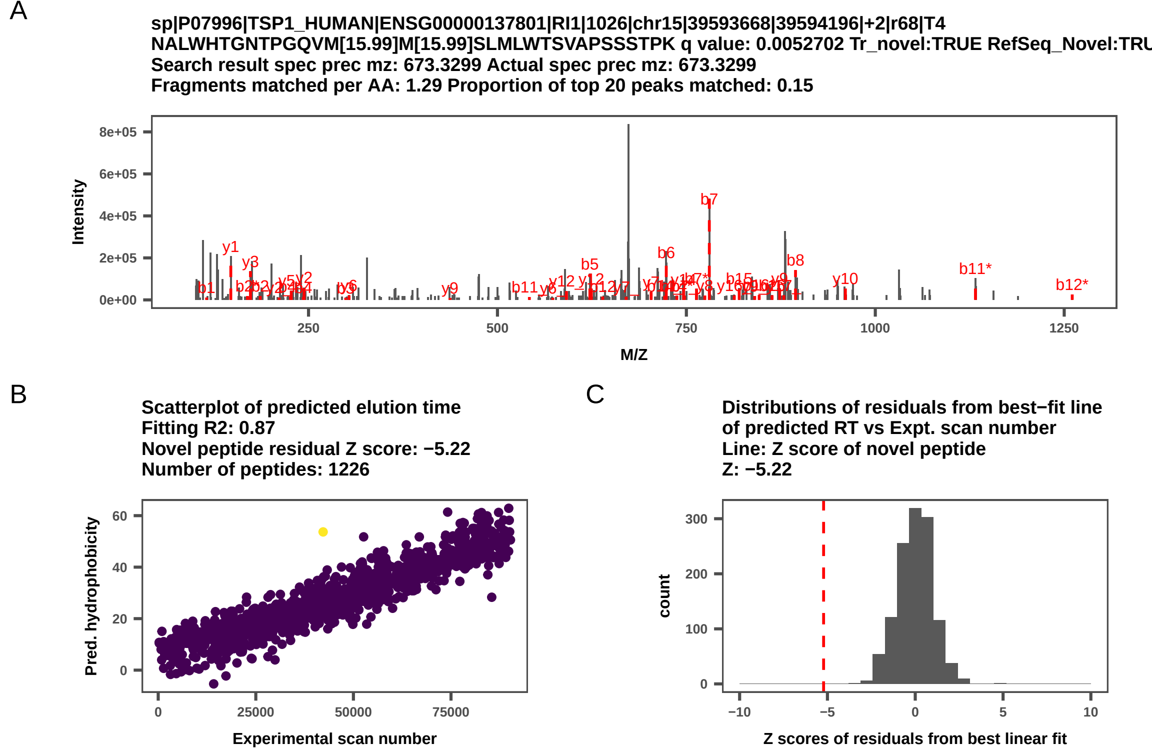
A
sp|P07996|TSP1_HUMAN|ENSG00000137801|RI1|1026|chr15|39593668|39594196|+2|r68|T4
NALWHTGNTPGQVM[15.99]M[15.99]SLMLWTSVAPSSSTPK q value: 0.0052702 Tr_novel:TRUE RefSeq_Novel:TRU
Search result spec prec mz: 673.3299 Actual spec prec mz: 673.3299
Fragments matched per AA: 1.29 Proportion of top 20 peaks matched: 0.15
Intensity
8e+05
6e+05
4e+05
2e+05
0e+00
M/Z
250
500
750
1000
1250
y1
y3
b1
b7
b6
b5
b8
b11*
b12*
y9
y10
b15
b11
B
Scatterplot of predicted elution time
Fitting R2: 0.87
Novel peptide residual Z score: −5.22
Number of peptides: 1226
Pred. hydrophobicity
60
40
20
0
0
25000
50000
75000
Experimental scan number
C
Distributions of residuals from best−fit line
of predicted RT vs Expt. scan number
Line: Z score of novel peptide
Z: −5.22
count
300
200
100
0
−10
−5
0
5
10
Z scores of residuals from best linear fit
